# Is now the time? Review of genetic rescue as a conservation tool for brook trout

**DOI:** 10.1002/ece3.10142

**Published:** 2023-05-25

**Authors:** Shannon L. White, Jacob M. Rash, David C. Kazyak

**Affiliations:** ^1^ U.S. Geological Survey Eastern Ecological Science Center Kearneysville West Virginia USA; ^2^ North Carolina Wildlife Resources Commission Marion North Carolina USA

**Keywords:** brook trout, conservation, evolutionary potential, genetic rescue, isolation

## Abstract

Brook trout populations have been declining throughout their native range in the east coast of the United States. Many populations are now distributed in small, isolated habitat patches where low genetic diversity and high rates of inbreeding reduce contemporary viability and long‐term adaptive potential. Although human‐assisted gene flow could theoretically improve conservation outcomes through genetic rescue, there is widespread hesitancy to use this tool to support brook trout conservation. Here, we review the major uncertainties that have limited genetic rescue from being considered as a viable conservation tool for isolated brook trout populations and compare the risks of genetic rescue with other management alternatives. Drawing on theoretical and empirical studies, we discuss methods for implementing genetic rescue in brook trout that could yield long‐term evolutionary benefits while avoiding negative fitness effects associated with outbreeding depression and the spread of maladapted alleles. We also highlight the potential for future collaborative efforts to accelerate our understanding of genetic rescue as a viable tool for conservation. Ultimately, while we acknowledge that genetic rescue is not without risk, we emphasize the merits that this tool offers for protecting and propagating adaptive potential and improving species' resilience to rapid environmental change.

## Graphical abstract

Many brook trout populations exist in small, isolated habitats where demographic stochasticity and the lack of gene flow threaten long‐term persistence. In this article, we review existing literature to evaluate whether genetic rescue may be a viable management goal for brook trout conservation.

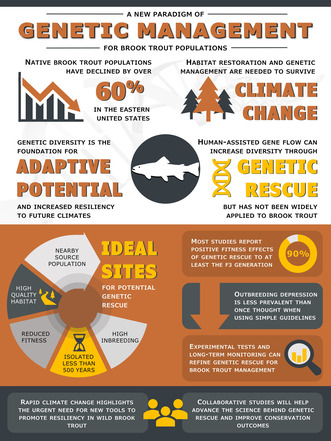

## 1 INTRODUCTION

Brook trout (*Salvelinus fontinalis*) is a widely distributed species of coldwater fish with a native range that spans throughout much of the eastern United States and Canada and non‐native, often invasive, populations in the western North America. Although brook trout were once abundant in many mountain streams and lakes, native populations have declined over the last century due to the interactive effects of habitat loss, climate change, and competition with non‐native species (Marschall & Crowder, 1996; McKenna et al., 2013; Stranko et al., 2008; Wagner et al., 2013). Brook trout are thought to have been extirpated or significantly reduced from over 60% of historical subwatersheds in the east coast of the United States (Hudy et al., 2008), which likely represents a conservative estimate of contemporary population loss given declines in brook trout vital rates observed over the last decade (Bassar et al., 2016).

Few large patches of suitable habitat remain for native brook trout in many regions of the United States, particularly in the species' southern range (Whiteley et al., 2013). Within these fragmented habitats, low carrying capacity coupled with extreme environmental stochasticity can result in significant interannual variation in abundance (Blum et al., 2018; Kanno et al., 2015; Letcher et al., 2007). Demographic stochasticity combined with limited effective connectivity can erode genetic diversity through genetic drift and inbreeding (Kazyak et al., 2022) and may reduce population vital rates and long‐term adaptive potential (Biebach et al., 2016). This interaction between environmental, demographic, and genetic processes may place many brook trout populations in extinction vortices (Gilpin & Soulé, 1986), where continued declines in population size and genetic diversity threaten long‐term species persistence across the landscape. Although some brook trout populations appear to be demographically secure (Huntsman & Petty, 2014), abundance may be a poor predictor of long‐term viability as populations of stable size can still experience cryptic declines in genetic diversity, contemporary fitness, and future adaptive capacity (Fraser et al., 2014).

Brook trout populations that have been recently (e.g., within 100 years) isolated by anthropogenic activities may be particularly vulnerable to demographic and genetic collapse, as bottlenecked populations frequently experience higher genetic loads owing to inbreeding, loss of genetic diversity, and increased prevalence of deleterious mutations (Keller & Waller, 2002). Isolated populations can persist on the landscape, but it may take thousands of generations to purge maladaptive alleles and accumulate beneficial adaptations needed for survival (Mathur et al., 2023). This may explain why some brook trout populations have persisted above natural barriers for several millennia, as sufficient evolutionary time has enabled populations to develop the genetic architecture needed for survival. However, in the absence of time, and with increasing environmental stochasticity providing unpredictable selection regimes, adaptation may be too slow to mitigate the negative effects of demographic and genetic stochasticity, placing recently isolated populations at high risk of extirpation.

Without natural connectivity to mitigate the demographic and genetic effects of isolation, the long‐term persistence of recently isolated brook trout populations could depend on active management strategies that target genetic conservation and restoration. One option may be genetic rescue, wherein the introduction of new alleles into a population results in increased genetic diversity, fitness, and adaptive capacity (Frankham et al., 2011; Whiteley et al., 2015). Genetic rescue occurs naturally among connected populations and is one mechanism responsible for the increased resiliency observed in large metapopulations (Hedrick & Garcia‐Dorado, 2016). However, in the absence of connectivity, human‐assisted migration can lead to similar patterns of transgressive hybridization and genetic rescue (Bell et al., 2019; Ralls et al., 2020).

Genetic rescue has been promoted as a potentially effective conservation tool, but much of the current understanding about the risks and benefits of genetic rescue is theoretical as implementation has largely been reserved for species of extreme conservation concern (Frankham, 2015; Ralls et al., 2018; Robinson et al., 2021) with only one documented case in wild brook trout (Robinson et al., 2017). Hesitancy to incorporate genetic rescue into management paradigms may reflect methodological uncertainty, with a lack of clarity about the potential risks and benefits of various implementation strategies. This also includes a tendency to conflate contemporary population stability with long‐term persistence probability, which can make it appear as though genetic management is not warranted for achieving long‐term conservation goals (Ralls et al., 2018).

Other reservations may stem from concerns about the potential harm that could result from outbreeding depression. This is a particular concern in brook trout, as the species shows remarkable levels of population differentiation at small spatial scales, with patterns of isolation‐by‐distance sometimes observed with sites separated by <1 km (Hudy et al., 2010; Kanno et al., 2011; Kelson et al., 2015). Divergent selection may result in strong patterns of local adaptation at fine spatial scales, with individuals showing significant, and sometimes unpredictable, differences in morphology, life history, physiology, and behavior (Ferchaud et al., 2020; Fraser & Bernatchez, 2005; Zastavniouk et al., 2017). This partitioning of genotypic and phenotypic diversity at small spatial scales may seem predictive of outbreeding depression and increase the potential risk associated with genetic rescue in brook trout conservation.

Although the scales of genotypic and phenotypic diversity may present a challenge for the application of genetic rescue in brook trout, we know that many populations are continuing to decline despite current conservation efforts (Hudy et al., 2008). Given projected climate regimes and the fundamental need for genetic diversity to allow for adaptation, we may now be at a stage where a more risk‐averse management strategy for many brook trout populations is active intervention through genetic rescue (Frankham, 2015). Moreover, brook trout could serve as a model species for experimental tests of genetic rescue that can help advance the science behind this important conservation tool. Few other species persist on the landscape as thousands of isolated populations, each of which representing an independent opportunity to test the eco‐evolutionary theories that are foundational to genetic rescue. High levels of genetic and phenotypic diversity and differentiation, along with a distribution shaped by geophysical and anthropogenic processes, are also likely to make decisions about where, and how, to perform genetic rescue dependent on the local context. This lack of a “one size fits all” approach to genetic rescue has not been addressed in the literature but is likely to be a more realistic representation of the concerns and constraints that many practitioners will face when evaluating the feasibility of a genetic rescue effort. As such, large‐scale, coordinated studies of the efficacy of different genetic rescue methods under a range of genetic and demographic scenarios in brook trout may be the pioneering effort needed for this tool to gain momentum in an era of conservation under rapid change and high uncertainty.

Our goal is to review existing theoretical and empirical studies to address some of the major uncertainties surrounding the use of genetic rescue in brook trout conservation. This manuscript is intended to be a practical guide that can be used by conservation practitioners when deciding if, and where, genetic rescue may be a successful management tool. Importantly, the purpose of this manuscript is not to universally recommend genetic rescue. Genetic rescue will most likely to be successful after careful consideration of the spatiotemporal factors shaping a population's genetic integrity, adaptive potential, and extirpation probability. Moreover, despite what the name implies, positive outcomes of a genetic rescue effort are not guaranteed. Genetic rescue has become a catchall term for human‐assisted gene flow, but we emphasize that by definition a population can only be considered rescued if it demonstrates a quantifiable increase in adaptive capacity following the introduction of new alleles (Bell et al., 2019; Frankham et al., 2011; Tallmon et al., 2004). As we discuss, many brook trout populations are unlikely to be ideal candidates for genetic rescue at this time. But, by coalescing information about the current status of genetic rescue, we hope to stimulate broader discourse that can assist conservation practitioners in determining the viability of genetic rescue in brook trout conservation planning.

## 2 GENETIC RESCUE—CURRENT KNOWLEDGE AND REMAINING UNCERTAINTIES FOR IMPLEMENTATION IN BROOK TROUT CONSERVATION

Because few have examined genetic rescue in wild or captive brook trout (but see Robinson et al., 2017; Wells et al., 2019), much of this review will be founded in theoretical studies and meta‐analyses of focal species that do not include lotic fishes. However, our goal is to not only present the scientific foundation of genetic rescue but also provide practical information for conservation practitioners when deciding if, and where, genetic rescue may be an appropriate management tool. Therefore, this manuscript is divided into major themes of uncertainty that have been expressed by brook trout managers concerning the application of genetic rescue, each of which we explore with a combined theoretical and practical perspective.

We also acknowledge that a unique challenge with the genetic management of brook trout is that it is a species of both conservation concern and significant recreational interest. Therefore, the future of genetic rescue in brook trout conservation planning may depend not only on the refinement of scientific protocols but also public and institutional opinions about the importance of genetic management of small, isolated populations. Accordingly, we discuss how changes in risk perception, specifically how individuals calculate the long‐term risk of genetic rescue compared with more traditional management approaches, may improve attitudes towards genetic management of isolated populations.

### 2.1 Is genetic rescue likely to benefit populations?

While many managers are familiar with the concept of genetic rescue, uncertainties about the relative risks and probability of success have limited its consideration in conservation planning. Early literature warned of the potential negative consequences of mixing formerly isolated populations, most notably the reduction in offspring vigor from outbreeding depression (Templeton, 1986; Thornhill, 1993). While outbreeding depression has been documented in some scenarios (e.g., Côte et al., 2014; Goldberg et al., 2005; Huff et al., 2011), more recent meta‐analyses suggest the threat can be minimal and negative outcomes are often predictable. For example, in a multitaxa study looking at the combined effects of outbreeding on fitness and survival, Frankham (2015) found that nearly 93% of 156 studies showed a positive effect of genetic rescue, with a median 148% increase in fitness of outbred populations occurring in wild or stressful environments. While many studies considered in the meta‐analysis by Frankham (2015) only measured fitness in the first generation, over a third of the included studies showed fitness benefits to the F2+ generation and a later study by Frankham (2016) found positive effects to at least the F3 generation. A meta‐analysis of wild and captive fishes by McClelland and Naish (2007) also found support for the positive fitness benefits of genetic rescue, although they noted the magnitude of the effect varied by taxa and response variable. Importantly, these meta‐analyses suggest that genetic rescue can have clear benefits for recently fragmented populations; however, it should be noted that they did not account for potential publication bias, and so it is possible that negative effects of genetic rescue were underreported in their analyses.

There has also been one case study of genetic rescue in wild brook trout by Robinson et al. (2017), who initially reported overall positive effects of outbreeding in four populations, with F1 hybrid offspring showing increased total length and outbred populations having higher genetic diversity (allelic richness and expected heterozygosity) compared with nonoutbred control sites. However, additional analyses on the F2+ generations revealed a more complex relationship between the degree of admixture and individual fitness (Robinson, 2022). That is, positive effects of outbreeding were only observed in individuals with intermediate levels of migrant ancestry. This result potentially highlights tradeoffs between outbreeding and local adaptation in isolated brook trout populations and suggests that the net effect of outbreeding may only be realized after several generations.

**FIGURE 1:**
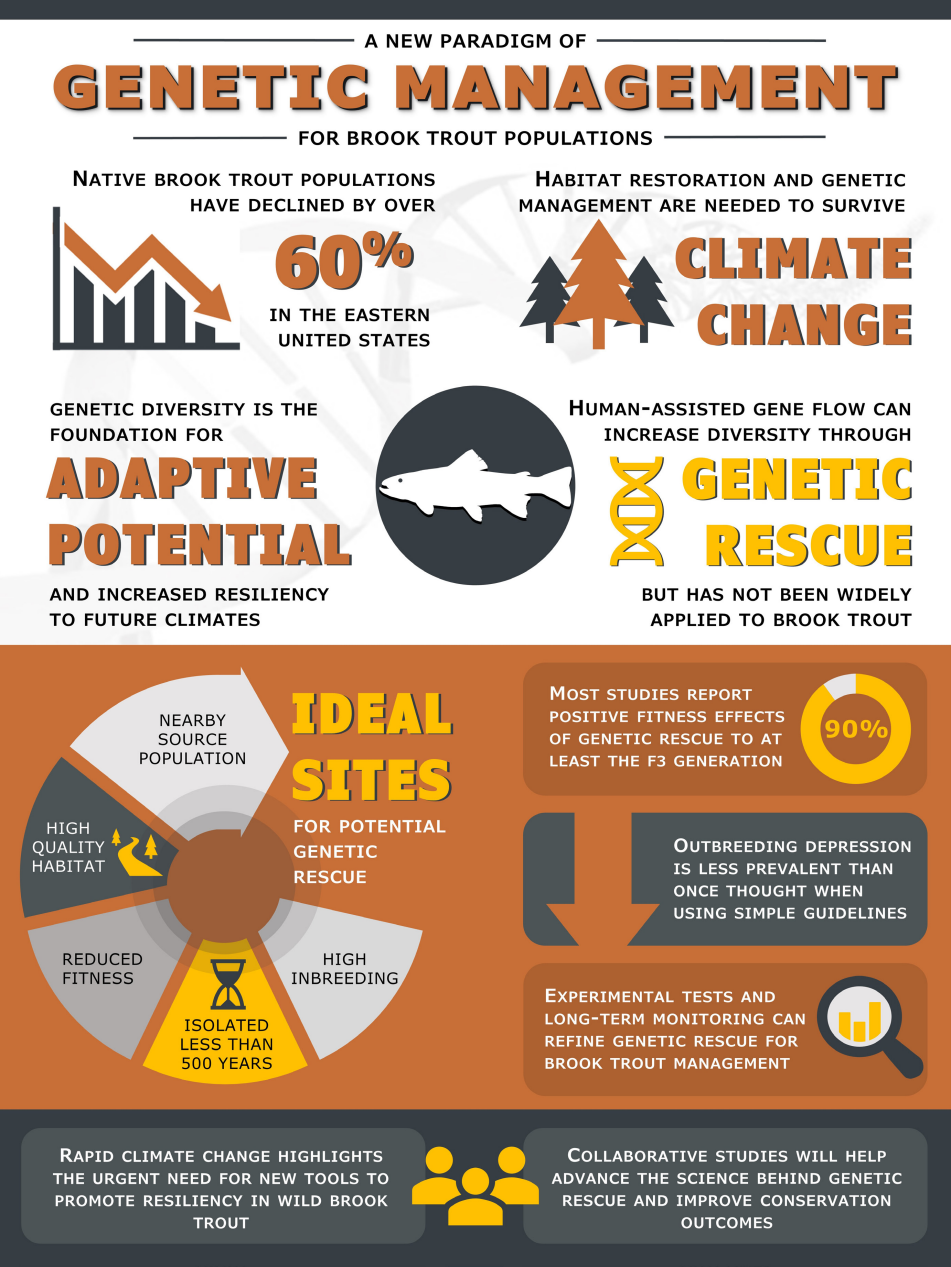
Given declines in brook trout populations and future climate change, genetic rescue may be an effective management strategy for the conservation of brook trout populations. Prior studies have identified design and monitoring protocols that may be effective for reducing the risk of outbreeding depression following genetic rescue. However, future collaborative studies may be efficacious for continuing to refine genetic rescue in stream fishes.

General success of genetic rescue efforts may be credited, at least in part, to clear guidance posited by Frankham et al. (2011) for the design of translocation efforts. Through a series of simulation analyses, the authors showed that outbreeding depression and widespread genetic homogenization can be minimized when translocation occurs between populations that have been separated by <500 years, occupy similar habitats, and show no fixed chromosomal differences (Figure 1). These guidelines have been widely adopted in subsequent genetic rescue attempts, and further refined as genomic methods have become available for assessing population structuring and quantitative genetic variation (Fitzpatrick & Funk, 2019).

However, as genetic rescue becomes more prevalent, it is also becoming apparent that some populations may still benefit from genetic rescue even when traditional design guidelines are not strictly followed. For example, Fitzpatrick et al. (2016) noted the demographic and genetic benefits of genetic rescue in populations of Trinidadian guppies (*Poecilia reticulata*) despite the use of an adaptively divergent source population. Likewise, Wells et al. (2019) found no consistent evidence of outbreeding depression in F1 brook trout that were propagated in captivity using gametes from wild populations that have been isolated for thousands of years. Additional work is needed to determine the long‐term fitness of hybrid offspring from highly divergent populations, but these initial studies provide some evidence that genetic rescue may be successful even when design guidelines are relaxed.

One significant shortcoming in our understanding of genetic rescue in any taxa is uncertainty about the expected duration of fitness benefits following translocation. Although positive effects of genetic rescue have been documented to at least the F3 generation (Fitzpatrick et al., 2020; Frankham, 2016), limited long‐term monitoring precludes more rigorous analyses of multigenerational fitness effects (Waller, 2015). This lack of information may be problematic, as reduced hybrid fitness is unlikely to be detected at the F1 generation, and so negative effects of genetic rescue may only become apparent in F2+ as recombination disrupts favorable gene complexes (Tallmon et al., 2004; Waller, 2015). In addition, genetic rescue success has traditionally been predicated on changes in phenotypic or demographic traits, with more recent studies also measuring changes in neutral genetic diversity. These traits are presumed to act as surrogates for fitness and adaptive capacity, but more rigorous genomic studies are needed to understand changes in evolutionary potential and quantitative trait frequency following outbreeding. Despite these uncertainties, early results suggest that when genetic rescue efforts are implemented in appropriate contexts, the probability of outbreeding depression is likely lower than the probability of extirpation due to inbreeding and drift (Frankham et al., 2011).

### 2.2 Where is genetic rescue most likely to be successful?

**FIGURE 2:**
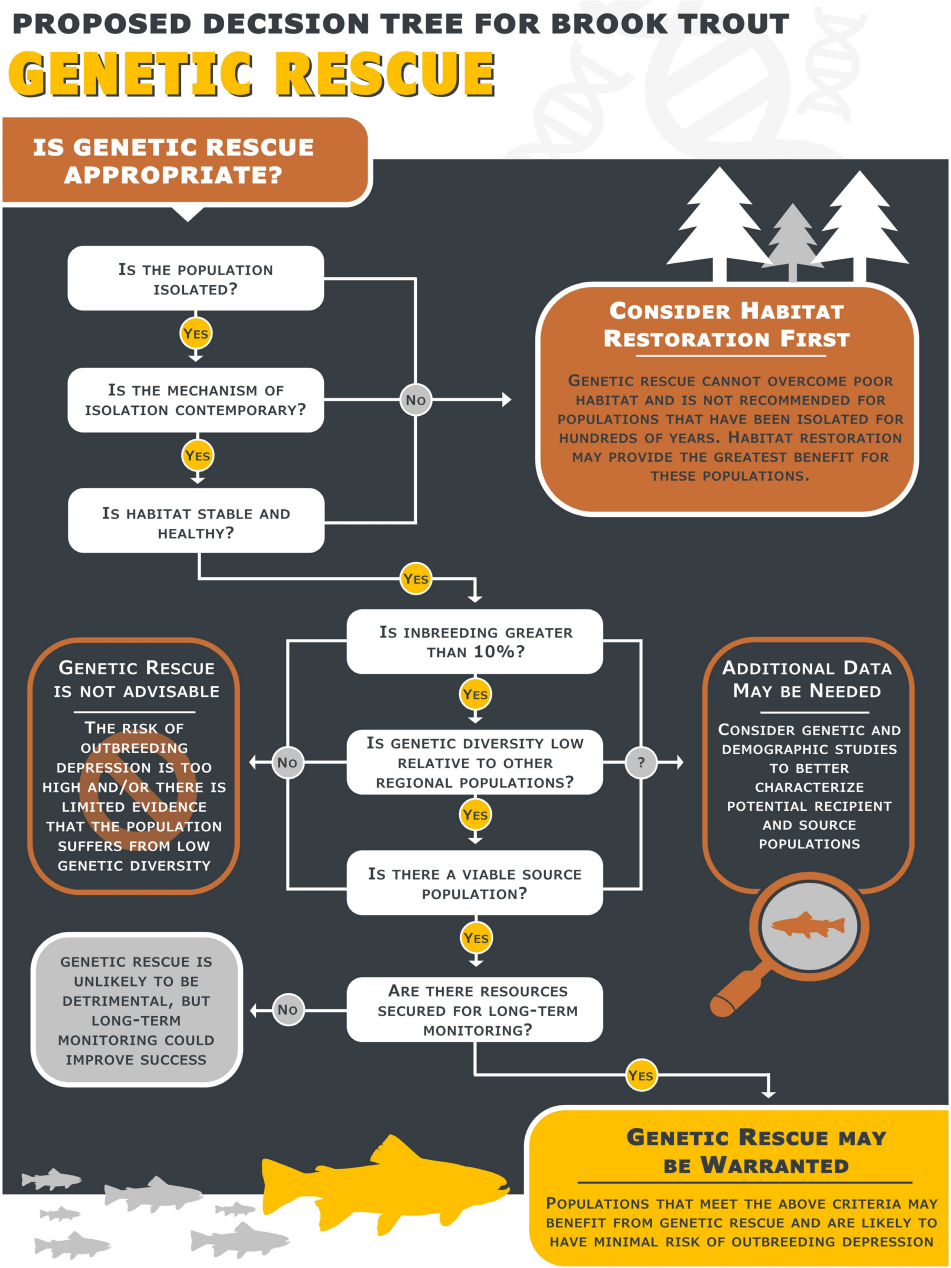
Following recommendations from prior studies, we present a proposed decision tree for determining whether a brook trout population is a potential candidate for genetic rescue. This decision tree can help users determine the suitability of genetic rescue for meeting conservation goals.

Isolation is a significant predictor of extirpation probability, yet paradoxically many brook trout populations have persisted above natural barriers for many centuries despite genetic isolation (Kelson et al., 2015; Torterotot et al., 2014; White et al., 2020). This highlights a significant shortcoming in our understanding of the demographic and genomic properties that underpin long‐term adaptive capacity and survival, and additional study is warranted to determine how these populations have survived despite the probable effects of inbreeding and genetic drift. In the interim, the persistence of many naturally isolated brook trout populations suggests these populations may not be the best candidates for genetic rescue. Moreover, the geophysical processes responsible for the isolation of these populations likely occurred >500 years ago, and so, the risk of outbreeding depression following the introduction of outside individuals may be high (Frankham et al., 2011). Studies have also shown that isolated brook trout populations can maintain high genetic diversity in large habitat patches (Whiteley et al., 2013), and long‐isolated populations are more likely to have developed important local adaptations and may have purged maladaptive alleles (Carim et al., 2017; Letcher et al., 2007; Stitt et al., 2014). As such, the conservation of brook trout populations with a long history of isolation may be best achieved through habitat modifications that are designed to improve population resiliency by increasing census and effective population size (Figure 2).

In contrast, brook trout populations that were isolated by more contemporary processes (e.g., anthropogenic movement barriers) and have lost substantial genetic diversity may be better candidates for genetic rescue. Ralls et al. (2018) suggested that genetic rescue efforts may be best focused on recently isolated populations that have an inbreeding coefficient of 0.1 and/or 10% reduction in genetic diversity. Populations that meet these criteria are more likely to have a greater risk of extirpation and see increases in fitness from even marginal increases in genetic diversity. Notably, census population size may be a poor criterion for evaluating the applicability of genetic rescue, as population size is not always correlated to genetic diversity and even populations at carrying capacity can still benefit from increased adaptive capacity that is expected to follow successful genetic rescue (Robinson et al., 2021).

Although genetic‐based criteria provide clear guidance for identifying candidate populations in need of genetic rescue, they may still be difficult to apply without historical reference data to demonstrate loss in genetic diversity over time or comparative data from other populations in the region. Even then, much of our current understanding about patterns of inbreeding, strength of genetic drift and selection, and patterns of isolation relies on analyses of putatively neutral microsatellites. While some have shown that microsatellites are effective for conservation prioritization (Farquharson et al., 2021; Hauser et al., 2021), recent developments in genomic methodologies are likely to improve our ability to detect populations that are at heightened risk of extirpation due to loss of adaptive variation. For example, using a reference genome for *Salvelinus* spp., authors have been able to disentangle the relative effects of neutral and adaptive genetic processes for shaping contemporary patterns of genetic diversity, ultimately providing insights into the colonization success of introduced brook trout populations (Brooks et al., 2022). Genomic marker panels specific to brook trout are currently in development and may enable similar studies that can help understand patterns of genetic structuring, effects of hatchery introgression, and sources of local adaptation (Meek & Larson, 2019). Accordingly, although microsatellite datasets offer a strong foundation for planning and executing genetic rescue efforts, marker panels that capture genome‐wide diversity may provide more opportunities to understand the population‐level response to rescue (Chen et al., 2022; Fitzpatrick & Funk, 2019; Whiteley et al., 2015).

### 2.3 How should genetic rescue be implemented?

Populations that are ideal candidates for genetic rescue not only demonstrate evidence of inbreeding and genetic diversity loss but should also be located in watersheds where there is a viable source population to use for translocation (Figure 2). While guidelines exist for identifying ideal source populations (Frankham et al., 2011), the suggested criteria may prove intractable for fishes of conservation concern, including many brook trout populations (Wells et al., 2019). The patchy, isolated distribution of extant brook trout populations engenders low genetic diversity and high divergence among many local populations (Kazyak et al., 2022), often to the extent that translocation may normally be considered inappropriate if applying traditional guidelines. However, recent studies have shown that genetic distance estimated from neutral microsatellite loci may be a poor predictor of outbreeding depression in brook trout (McClelland & Naish, 2007; Wells et al., 2019), and that admixture between divergent populations can still result in heterosis (Kronenberger et al., 2018; Tallmon et al., 2004). Moreover, neutral genetic differentiation is typically much greater in populations with low diversity, presumably as a result of random loss of alleles through drift (Kazyak et al., 2022). This suggests that, while genetic differentiation is important to consider in source population selection, it will likely be prudent to consider this criterion alongside other genetic diversity metrics. For example, provided source and recipient populations are phenotypically similar, translocating individuals from larger, more genetically diverse populations may provide a more substantial increase in genetic diversity and fitness than would be expected if migrant alleles originated from a smaller, inbred source (Ralls et al., 2020). However, a “bigger is better” approach to source population selection may not always be advantageous, as heterozygotes in large, genetically diverse populations are expected to carry recessive deleterious mutations. Following population contraction, as would occur when randomly sampling individuals for translocation, and subsequent inbreeding in an isolated population, the prevalence of the deleterious alleles may then increase causing a rise in genetic load that could lead to population collapse (Kyriazis et al., 2021). Together, this suggests that source population selection is likely to be optimized when census size, standing genetic diversity, and population differentiation are all balanced in the decision‐making process.

Most previous genetic rescue efforts have employed a one‐way transfer of individuals from a single source population to one recipient population (Tallmon et al., 2004). While this design might be appropriate when the number of populations in need of genetic rescue is minimal, it may not be the most efficacious for species like brook trout where many local populations could benefit from human‐assisted gene flow. Under these circumstances, reciprocal transfer between two or more populations could provide an opportunity for simultaneous genetic rescue of multiple populations (Heber et al., 2013). Because there is no net change in demographic structure with the reciprocal transfer, this approach may have the added benefit of reducing the probability of Allee effects that could incur following the reduction in the size of the source population (Dennis et al., 2016).

Another option for reducing the demographic risk of removal on source populations is to translocate individuals from multiple source locations and/or across multiple years (Weeks et al., 2017; Weise et al., 2020). Translocating fish from multiple sources can result in a greater increase in genetic diversity and fitness in the recipient populations but may incur a higher risk of outbreeding depression through genetic swamping or genetic homogenization of regional populations. Alternatively, the sequential transfer of relatively few individuals from the same population, but across multiple years, could minimize the potential for stochastic processes to negatively affect the outcome of genetic rescue in both source and recipient populations.

Fewer guidelines exist for determining the number of individuals that should be translocated in each genetic rescue attempt. This may be because the optimal number is likely to be context‐specific and dependent on the level of inbreeding and the effective size of both source and recipient populations (Frankham, 2015). Under idealized conditions, reinstating one effective migrant per generation would create sufficient connectivity to minimize the loss of diversity and divergence due to genetic drift (Mills & Allendorf, 1996). However, translocating one individual per generation has higher reoccurring resource costs that may be difficult to secure at the onset of the project. In addition, one effective migrant per generation can lead to more outbreeding depression than a single translocation event that results in 50% migrant individuals (Edmands & Timmerman, 2003). Therefore, a single transfer of more individuals is often an appealing design for genetic rescue, with Frankham (2015) suggesting a general rule that migrant alleles should be limited to <50% of the population to avoid genetic swamping.

One challenge with identifying the number and source of individuals to translocate is that genetic rescue ultimately depends on successful interbreeding between source and recipient populations. Guidelines that stress recent connectivity and phenotypic similarity between populations are designed to maximize admixture by reducing the probability that reproductive isolation will limit mating success. While spawning phenology has been identified as one barrier to admixture in brook trout (Kulp et al., 2017), pre‐ and post‐zygotic reproductive barriers can still be difficult to predict. Moreover, bias in reproductive success among migrant and resident individuals is likely to occur (White et al., 2022) and can significantly affect the extent to which migrant alleles are transferred to the resident population. As a result, realized genetic diversity following rescue is likely to be less than the theoretical maximum diversity that would occur with even and complete admixture.

The aforementioned uncertainties about implementation strategies underscore genetic rescue as a nascent management tool that involves complex evolutionary processes. Our current understanding of genetic rescue is particularly limited by the short duration of monitoring efforts, with most previous studies evaluating project success after only a few reproductive cycles (Robinson et al., 2021). Limited monitoring can be problematic because the power to detect positive fitness effects of admixture is often lowest in the first few generations (Robinson et al., 2021) and the effects of genetic rescue on diversity and fitness may not be constant or linear over time. For example, hybridization between divergent source populations can increase the prevalence of maladaptive traits and lower population genetic variation and fitness in any one generation. However, in later generations, admixture can result in new genetic combinations and phenotypes that facilitate expansion into new ecological niches, thereby increasing overall population resiliency (Derry et al., 2019; Whiteley et al., 2015). Because the effects of genetic rescue will vary over multiple temporal scales, future efforts may benefit from long‐term, multimetric genetic and demographic evaluations (see Robinson et al., 2021 for a discussion on the utility and statistical power of commonly used evaluation metrics).

### 2.4 What resources are needed for implementation of a genetic rescue program?

While genetic rescue is likely to be cheaper and can incur more long‐term fitness benefits than physical habitat manipulation alone (Frankham, 2015), resource planning for genetic rescue efforts is still likely to present a challenge to implementation. Costs associated with the collection and analysis of genetic data can be significant (Taft et al., 2020). Additionally, while genetic data can be used to answer diverse research questions, it can be difficult to justify their value over more tangible habitat restoration projects. As such, there may be little financial or institutional support for long‐term, comprehensive monitoring programs that are critically needed to evaluate project success and refine genetic rescue protocols.

The most resource‐intensive element of a genetic rescue effort is likely to be the genetic data themselves. Baseline data are important for identifying populations that are ideal candidates for rescue (i.e., inbred with declining genetic diversity and census and effective population sizes and a recent history of isolation). Likewise, regional population genetic analyses are necessary to identify source populations with enough genetic diversity to enable genetic rescue and to screen for introgression with domestic hatchery lineages (e.g., Kazyak et al., 2021). This highlights the benefits of genetic monitoring programs that maintain population genetic data and can be quickly referenced for project planning. Some states have implemented widespread genetic monitoring programs for brook trout, most notably North Carolina, which maintains a genetic database of nearly 500 brook trout populations in the state (Kazyak et al., 2021). However, given that genetic rescue efforts are likely to be implemented at more local levels, concentrated studies at smaller spatial scales will likely provide equally sufficient data. In the future, declining costs associated with the production of genetic and genomic data are poised to make the development of necessary datasets more feasible for managers and conservation practitioners (Whiteley et al., 2015).

### 2.5 Has active genetic management been undervalued in present‐day conservation?

The field of conservation genetics has matured in recent decades, but some argue that it remains an academic discipline that has been slow to incorporate into applied species management (Taylor et al., 2017). While many practitioners agree on the importance of considering genetic processes in conservation planning (Kardos et al., 2021), there can be a significant gap between data presented in academic reports and the information that is needed to integrate genetics into species management plans (Taylor et al., 2017). This “conservation genetics gap” is further compounded for newer tools like genetic rescue, where much of the theoretical framework is still under development and there are relatively few empirical applications that exemplify success. Others have also noted that hesitancy may be a byproduct of academic training programs that have traditionally emphasized managing for isolation to preserve genetic uniqueness, with little regard for historical patterns of population connectivity or the evolutionary processes that maintain populations and allow continued adaptation to their environment (Ralls et al., 2018).

In addition to scientific uncertainty, genetic rescue may be one of relatively few existing management strategies that explicitly aims to alter population genetic structure, and so, it can be perceived as radical, and potentially unjust (Kronenberger et al., 2018; Ralls et al., 2018). However, many traditional management tools such as hatchery stocking, size‐specific harvest restriction, and habitat alteration are also known to affect genetic processes (Ralls et al., 2018), yet their application has previously been less contentious because they are used to meet more traditional management goals focused on population demographics and improved recreational opportunities. Conventional fish management interventions are also more readily observable by the general public and exhibit benefits over short time scales. While stakeholder perspectives remain diverse, there is growing recognition of both the importance of genetic diversity and the reality of environmental change (Klütsch & Laikre, 2021). Recent population losses in many areas (Hudy et al., 2008) underscore the value of complementing traditional management strategies with additional tools to promote adaptation to changing conditions.

This diversity in stakeholder interests scales to many fisheries managers, who may be confronted with a decision about whether to allocate limited resources towards management approaches that bolster present‐day populations or more novel paradigms such as genetic and/or evolutionary rescue that seek to balance contemporary population security with longer‐term genetic management. This choice may be challenging, as conventional management strategies may be considered low‐risk because they have prescriptive guidance for implementation, reasonably predictable outcomes that can be readily documented, and well‐understood expectations from stakeholders (Ralls et al., 2018). However, traditional fisheries management techniques have been insufficient to halt or reverse landscape‐scale declines in occupancy (Hudy et al., 2008) and have also been inadequate to maintain or improve genetic diversity (Kazyak et al., 2022). Therefore, although genetic rescue carries risk and uncertainty, it has the potential to fill a significant void in our current management capabilities. Moreover, in the highly fragmented landscapes that are pervasive across much of the range of brook trout, genetic rescue is one of the only approaches that can provide the genetic variation needed for future adaptation to changing conditions.

## 3 IS NOW THE TIME TO INCORPORATE GENETIC RESCUE INTO BROOK TROUT CONSERVATION?

Genetic rescue has traditionally been used as a tool of desperation, rather than an antidote for the effective management of small, isolated populations (Robinson et al., 2021). However, as vulnerable populations continue to decline and become extirpated, many are starting to reconsider the conditions under which genetic rescue may be appropriate. For example, Frankham (2015) argued that criteria previously established for populations in need of genetic rescue—namely significant inbreeding and loss of fitness—were not only too stringent but also not identifiable in most populations. This, along with mounting evidence that outbreeding depression is largely avoidable, provides more support for the use of genetic rescue (Frankham et al., 2011; Kovach et al., 2022; Wells et al., 2019) to minimize further population declines attributable to overly cautious management approaches.

Theoretical and experimental studies have greatly advanced our understanding of the ideal conditions and expected outcomes of genetic rescue efforts (e.g., Frankham et al., 2011). As noted, genetic rescue is unlikely to be an ideal conservation strategy for historically isolated populations that are demographically and genetically stable. However, for those populations that are experiencing precipitous declines, there may be a tendency to allow lingering uncertainty to inhibit more active conservation strategies. While it is prudent to recognize remaining knowledge gaps, the reality is that we may never have the data needed to disentangle the complicated ecological and evolutionary processes that determine project success (Whiteley et al., 2015). Risk will always be a factor in genetic rescue efforts, but the crisis discipline of conservation biology often demands action and decision amid high uncertainty and incomplete information (Gregory et al., 2013; Soulé, 1985).

Such risk and opportunity highlight the potential benefits of experimental application and evaluation of genetic rescue. Although brook trout is a species of conservation concern across much of their native range in the United States, it also represents an ideal taxon for theoretical and empirical tests of genetic rescue. Many watersheds with vulnerable brook trout populations share a history of contemporary anthropogenic disturbance, which resulted in the isolated, patchy distribution of numerous populations that are threatened by increasing genetic loads from inbreeding and genetic drift (Hudy et al., 2008; Kazyak et al., 2022; Pregler et al., 2018). The large number of isolated brook trout populations within relatively small geographic regions presents a good opportunity to investigate the efficacy of different study designs and address key uncertainties about the best methods for implementing genetic rescue. Moreover, because isolated populations lack connectivity, any potential adverse outcomes should be restricted to the experimental populations.

However, the longer adaptive genetic management is delayed, the more difficult it may be to achieve positive outcomes of genetic rescue efforts. As the number and genetic diversity of extant populations continue to erode, genetic rescue may require the translocation of an increased number of individuals from more distantly located sources—both of which would entail a greater risk of outbreeding depression and genetic homogenization across larger spatial scales. Moreover, the success of the genetic rescue is predicated on the restoration of genetic diversity so as to increase adaptive capacity. As endemic diversity is lost, it may be impossible to recover sufficient variability needed for natural selection to generate adaptive traits (Perrier et al., 2017). Even with advancements in genomic technologies, it is unlikely that we will be able to predict the most advantageous genotypes for securing long‐term population survival as adaptive traits (e.g., thermal tolerance, body morphology, bioenergetics, etc.) are often polygenetic traits with spatially varying genomic architectures. Therefore, it may be more effective to use approaches like genetic rescue and reintroduction to preserve contemporary genetic diversity and allow important genes to persist on the landscape.

While genetic rescue appears to be a promising tool to support brook trout management, we are reminded that a guiding principle of conservation biology is to first do no harm (Gregory et al., 2013). Uncertainty remains, and future efforts will benefit from thoughtful study designs that clearly articulate goals and perceived obstacles to success and incorporate multimetric genetic and demographic data in planning and monitoring. Fastidious application and documentation are particularly critical in future genetic rescue attempts so that experiments can benefit from learned knowledge. Although these studies are poised to rapidly progress our understanding of best management principles for genetic rescue, early failure could cast premature doubt on the viability of this important management tool. For this reason, range‐wide, collaborative efforts may be particularly beneficial for rapid data synthesis and to understand the efficacy of genetic rescue under a range of demographic and genetic scenarios. For example, well‐designed common garden experiments conducted across latitudinal gradients may be able to disentangle the relative effects of population size, genetic diversity and differentiation, patch size, and years since isolation in determining the probability of successful genetic rescue. It may also be possible to quantitatively test the efficacy of different methodologies to optimize the number and age class of translocated individuals. These concurrent efforts may allow for more powerful metanalysis on the efficacy of genetic rescue, while minimizing risk and resource costs to any specific region.

## 4 CONCLUSIONS

Given past declines and ongoing stressors, many brook trout populations are likely on a trajectory towards extirpation. Contemporary anthropogenic processes have left many populations in small, isolated fragments of habitat, where lack of gene flow could risk declines in population fitness and survival through loss of genetic diversity and inbreeding depression. Although many regions have yet to experience widespread extirpation events, short‐term demographic stability does not guarantee long‐term population viability. For many populations, persistence under future climate change scenarios is likely contingent on the capacity for adaptation, which is a function of standing genetic variation. If our management goal is to ensure the long‐term survival of brook trout populations, then genetic rescue through human‐assisted gene flow may be an effective strategy to promote adaptive capacity in anthropogenically isolated populations.

Implementation of genetic rescue is not without risk. However, the continued loss of brook trout populations reflects the inherent gamble of current management paradigms. Genetic rescue may provide a viable alternative that could help reduce the rate of contemporary brook trout extirpation and promote resilience to future environmental change. As such, with additional experimental implementation and refinement of protocols, genetic rescue may be a promising management tool for the conservation of isolated brook trout populations.

## AUTHOR CONTRIBUTIONS

**Shannon L. White:** Conceptualization (equal); investigation (lead); visualization (lead); writing – original draft (lead); writing – review and editing (lead). **Jacob M. Rash:** Conceptualization (equal); supervision (equal); writing – original draft (supporting); writing – review and editing (supporting). **David C. Kazyak:** Conceptualization (equal); supervision (supporting); writing – original draft (supporting); writing – review and editing (supporting).

## FUNDING INFORMATION

None.

## CONFLICT OF INTEREST STATEMENT

The authors declare no conflicts of interest.

## Data Availability

Data sharing not applicable to this article as no datasets were generated or analysed during the current study.
